# Ultrasound-guided central venous line placement in critically ill patients: is chest X-ray needed to assess post-insertion pneumothorax?

**DOI:** 10.1186/cc10818

**Published:** 2012-03-20

**Authors:** O Samir Abdel Gelil Kotb, A Ali Abel Aziz, Y Awad

**Affiliations:** 1 Sheikh Khalifa Medical City, Abu Dhabi, United Arab Emirates

## Introduction

Critically ill patients, mostly on positive pressure ventilation, are at higher risk of pneumothorax as well as their need for a central venous line (CVL) to optimize fluid status, CVP measurement, and so forth, and where the CVL is not being placed in the best circumstances with the patients being critically ill, unstable and with higher chances of error predisposed by pre-existing lung disease, obesity or whatever the admitting diagnosis. Before CVL placement was a blind technique relying on the anatomical positions identifying the position of major blood vessels and thus post-insertion X-ray was needed to confirm correct placement and to assess for pneumothorax. But with ultrasound (US) being more widely available, and most CVLs placed as US guided, the ultimate question develops: is post-insertion chest X-ray still needed?

## Methods

A retrospective study of 856 lines placed in 602 patients being evaluated over a period of 11 months. All cases were performed in a controlled ICU environment. Chest X-rays were performed 30 minutes post-insertion in the D0 adult ICU unit in a tertiary medical center in Abu Dhabi, UAE. The D0 ICU has a capacity of 24 beds with an average admission rate of 55 to 60 patients per month. Records were assessed and evaluated, and data collected and statistically studied.

## Results

A total of 856 lines performed in 602 patients were evaluated. In 607 US-guided cannulating internal jugular veins with only four cases of malposition, there were no cases of pneumothorax recorded. A total of 161 subclavian veins were cannulated with no US, of which six cases of pneumothorax were reported; two cases needed intercostal tube insertion. Eighty-eight femoral vein cannulations with no US were performed and no complications were recorded.

## Conclusion

Chest X-ray is not necessary after US-guided CVL placement. Cutting out the chest X-ray procedure post insertion proved to be cost-effective.
